# ALK and IGF-1R as independent targets in crizotinib resistant lung cancer

**DOI:** 10.1038/s41598-017-14289-w

**Published:** 2017-10-24

**Authors:** Christabel Wilson, Mhairi Nimick, Hayley Nehoff, John C. Ashton

**Affiliations:** Department of Pharmacology & Toxicology, Otago School of Biomedical Sciences, University of Otago, Dunedin, New Zealand

## Abstract

ALK positive non-small cell lung cancer is highly responsive to ALK inhibitors such as crizotinib, but drug resistance typically develops within a year of treatment. In this study we investigated whether IGF-1R is an independent druggable target in ALK-positive lung cancer cells. We confirmed that combination ALK and IGF-1R inhibitor treatment is synergistically cytotoxic to ALK-positive lung cancer cells and that this remains the case for at least 12 days after initial exposure to crizotinib. ALK-positive cells with acquired resistance to crizotinib did not acquire cross-resistance to IGF-1R inhibition, though combination treatment in the resistant cells gave additive rather than synergistic cytotoxicity. We concluded that IGF-1R is an independent druggable target in ALK-positive lung cancer and support the trial of combination treatment.

## Introduction

Non-small cell lung cancer (NSCLC) causes approximately 80–85% of lung cancer deaths^1,2^ and mutations associated with the anaplastic lymphoma kinase (ALK) gene occur in 3–8% of lung cancer patients^3,4^. Crizotinib is an ALK inhibitor that was approved by the Food and Drug Administration in 2011 for the treatment of advanced ALK-positive (ALK^+^) NSCLC (6–9). ALK^+^ lung cancer patients have an objective response rate (ORR) to crizotinib of 60.8% and a progression free survival time (PFS) of 9.7 months^5–9^. However, patients who initially respond to crizotinib develop drug resistance, typically within one year of treatment.

Various mechanisms of crizotinib resistance have been investigated^10–13^, including increased activation of IGF-1R, which has been proposed as a tyrosine kinase bypass signalling pathway. Lovly *et al*.^14^ showed that combining IGF-1R with ALK inhibitors could overcome resistance in crizotinib resistant ALK^+^ lung cancer cells *in vitro*. Along with the clinical observation that an ALK^+^ lung cancer patient responded dramatically to IGF-1R inhibition, this led the authors to propose the trial of dual inhibition of ALK and IGF-1R in ALK^+^ lung cancer patients; justified as a strategy to delay or overcome the development of crizotinib resistance. The authors also proposed that the efficacy of ceritinib against crizotinib resistant lung cancer might be partially explained by its dual IGF-1R/ALK inhibitory action.

We tested these hypotheses further by developing our own *in vitro* models of crizotinib resistance using human ALK^+^ lung adenocarcinoma cells, studying both the effects of IGF-1R inhibition shortly after crizotinib treatment and chronic crizotinib exposure. A secondary aim of our experiments was to assess whether ALK and IGF-1R represent independent drug targets according to the criterion for combinatorial drug treatment developed by Bozic and Nowak^15,16^ such that drug resistance mechanisms for the two targets are independently distributed among cancer cell clones. By studying both the effects of short term and long term crizotinib treatment on ALK^+^ NSCLC cells to IGF-1R sensitivity we aimed to determine the independence of ALK and IGF-1R as drug targets both with respect to innate and acquired crizotinib resistance. Our results support the trial of combinational treatment.

## Materials and Methods

### Cell culture

All cell lines were maintained in a humidified incubator 5% CO2 at 37 °C. Human lung adenocarcinoma cell line A549 (harbouring a KRAS gene codon 12 point mutation) were used as a comparison cell line, and maintained in RPMI1640 media (ThermoFisher, US) supplemented with 2% foetal bovine serum (FBS, Sigma-Aldrich, NZ), and ELM4-ALK mutated H3122 cells incubated in RPMI1640 media with 5% FBS. Both cell lines were maintained in 1% penicillin/streptomycin (100 μg/mL) (Sigma-Aldrich, AU).

### Drugs

Crizotinib and ceritinib, NVP-AEW541 (NVP) (LC Laboratories, US) and AZD3463 (ApexBio Technology LLC, US) were dissolved in 0.1% DMSO (Sigma, AU) for all experiments.

### Generation of crizotinib-resistant H3122 cells

Innate resistance to crizotinib was studied by treating H3122 cells with a 10 μM of crizotinib for 24 hours and then replacing the media without crizotinib. Remaining cells were studied over 12 days following crizotinib treatment. We refer to the cells treated in this way as 24-H3122. To generate a cell line with acquired crizotinib resistant, parental H3122 cells were cultured with increasing concentrations of crizotinib starting with 0.40 μM for 24 hours, increased to 0.56 μM on day 2, and 0.80 μM on day 3. Media was changed every 3 days supplemented with fresh drug. A stable crizotinib resistant cell line was developed after 4 months of culturing in the presence of the drug and termed CR-H3122.

### Cell proliferation and growth assays

Cells were seeded in 96-well plates at a density of either 7000 cells (H3122, 24-H3122 and CR-H3122) and 4000 cells (A549) and allowed to adhere for 24 hours. Cells were treated with individual drugs alone or drug combinations for 72 hours before assay. Cytotoxicity and proliferation rate was evaluated using the sulforhodamine B (SRB) assay, as described by Skehan *et al*. (1990). Briefly, cells were fixed with 50 µL 10% trichloroacetic acid (TCA) for 30 minutes at 4 °C. Protein was stained with 50 µL of SRB and then the wells washed with 1% acetic acid. The plate was then dried and SRB solubilised in 100 µL of 100 μM TRIS buffer (Sigma-Aldrich, US). Absorbance was read at 490 nm with a Spectromax plate reader, deducting the background of 630 nm; cell growth inhibition was evaluated as the ratio of the absorbance of the treated cells with the DMSO-treated control. All cytotoxicity assays were carried out in three independent experiments measured in technical triplicate. Cell proliferation time points were measured in hextuplicate.

### Cell cycle analysis

To assess the effect of crizotinib on the cell cycle in our model of innate resistance H3122 cells were seeded in a 6-well plate at a density of 10,000 cells per well and incubated overnight to allow for cells adhesion prior to treatment. Cells were treated with crizotinib (10 μM) for 24 hours after which cells were washed with 0.01 M Phosphate buffered Saline (PBS, Sigma-Aldrich US) and lysates collected via centrifugation and fixed with 70% ethanol over the following 12 days. For cell cycle analysis, cells were washed with 0.01 M PBS, treated with RNase (20 mg/mL), and then stained with propidium iodide (PI, Sigma-Aldrich, US). Cells were processed with the GalliosTM flow cytometer (Beckman Coulter, Life Sciences, Indianapolis, USA) and analysed with FlowJo LLC version 10 (FlowJo LLC, Ashland, Oregon, USA). Three independent experiments were carried out measured in technical triplicate.

### Immunoblotting

For Western blot assays cells were first seeded at a density of 200,000 cells per well in 6 well plates. Following treatments cells were harvested, washed in 0.01 M PBS and lysed in a buffer consisting of 50 μM Tris-HCl (pH 8), 150 mM NaCl, 1 μM EDTA, 1 mM NaF, 1 mM sodium orthovanadate, 1 mM phenylmethylsulfonyl fluoride (PMSF), 1% TritonX-100, and 1% SDS. Cell extracts were clarified by centrifugation (12,500 RPM at 4 °C for 8 mins) and lysates then subjected to SDS-PAGE followed by transfer to a PVDF membrane which was probed for proteins of interest with primary antibodies, diluted in 0.01 mM PBS with 1% Bovine serum albumin (BSA, Life Technologies NZ). Primary antibodies used were for: ALK (D5F3) (1:2000), phospho-ALK (Tyr1507) (1:1000), IGF-1 Receptor β (D2H3) (1:2000); phospho-IGF-1R β (Tyr1134/1136) (1:1000), SRC (2123) (1:1000). phospho-SRC (Tyr416) (6943) (1:1000), Goat anti-rabbit HRP conjugate (7074) (1:2000), (Cell Signaling Technology (MA, USA); ERK (M5670) (1:5000) phospho-ERK1&2 (M9692) (1:1000), β-tubulin (T5T43) (1:1000), (Sigma, US); Goat anti-mouse HRP conjugate Rabbit mAb (1:1000) (US1401253) (Merk Millipore, US).

### Data analysis

Cytotoxicity data were log transformed, normalised to control, and analysed using a 4 parameter nonlinear regression model. Differences in IC_50_ values were assessed using the extra sum of squares test where the null hypothesis is the global model with shared IC_50_ values (Graphpad Prism 7 software). Where appropriate, data were analysed with one-way ANOVA, followed by Bonferroni post hoc tests. Cell cycle data were normalised to vehicle control (0.1% DMSO) and analysed using a two-way ANOVA coupled with a Bonferroni post-hoc test. Statistical significant results were considered to be when p < 0.05. The Chou-Talalay combination index (CI) method was used^17,18^ to measure drug synergy, (Supplementary material). Cells were treated with concentrations of crizotinib (0.025, 0.05, 0.10, 0.50, 1 μM) and NVP (0.5, 1, 2, 3, 4.5 μM) as single agents and in combination after which cell viability was assessed using the SRB assay.

## Results

**Figure 1:**
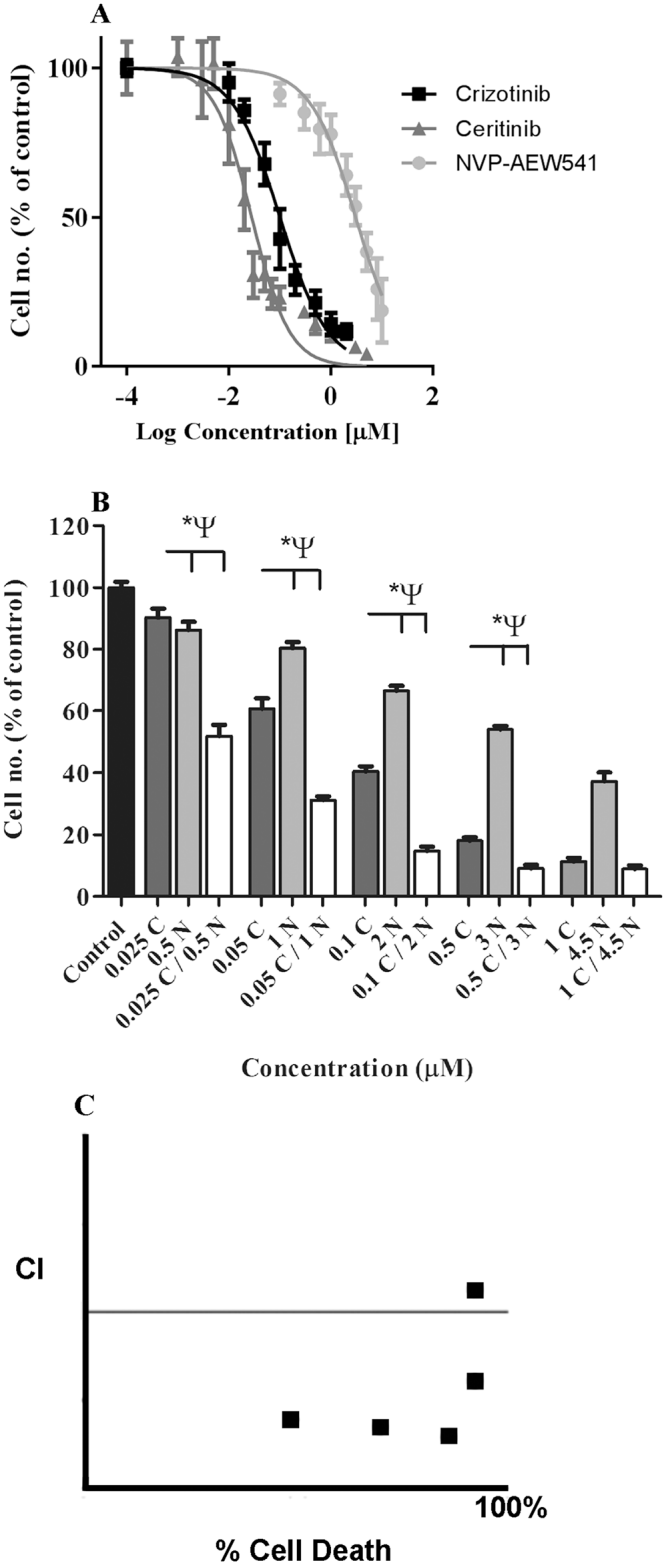
Effect of combination ALK and IGF-1R inhibition on ALK + H3122 lung cancer cells. (**A**) H3122 cell cytotoxicity of ALK inhibitors crizotinib and ceritinib, and IGF-1R inhibitor NVP-AEW541, data points are means and error bars are sd. (**B**) H3122 cell cytotoxicity of combinations of crizotinib and NVP-AEW541. Black bar is DMSO control, dark grey bars crizotinib alone, light grey bars NVP-AEW541 alone, and clear bars are combination of both. Error bars are SEM.(**C**) Combination index plot for drug combinations in “B”. The horizontal line represents additivity, above the line inhibition, and below the line synergy. All data represent three independent experiments carried out in triplicate. *p < 0.05 for crizotinib vs. combination, ^Ψ^p < 0.05 for NVP-AEW541 vs. combination.

We first tested the effects of ALK inhibition in combination with IGF-1R inhibition in untreated H3122 cells. IC_50_ values for ALK inhibitors crizotinib and ceritinib, and the IGF-1R inhibitor NVP-AWE453 (NVP) were 0.03 ± 0.06, 0.03 ± 0.03, and 2.99 ± 0.05 μM respectively (mean ± SEM, n = 3) (Fig. 1A). Combination of crizotinib with NVP produced strong toxicity (Fig. 1B); Loewe combination indices showed that all except the lowest concentrations of the drugs were strongly synergistic in combination (Fig. 1C), consistent with previous reports by Lovly *et al*.^14^.

**Figure 2:**
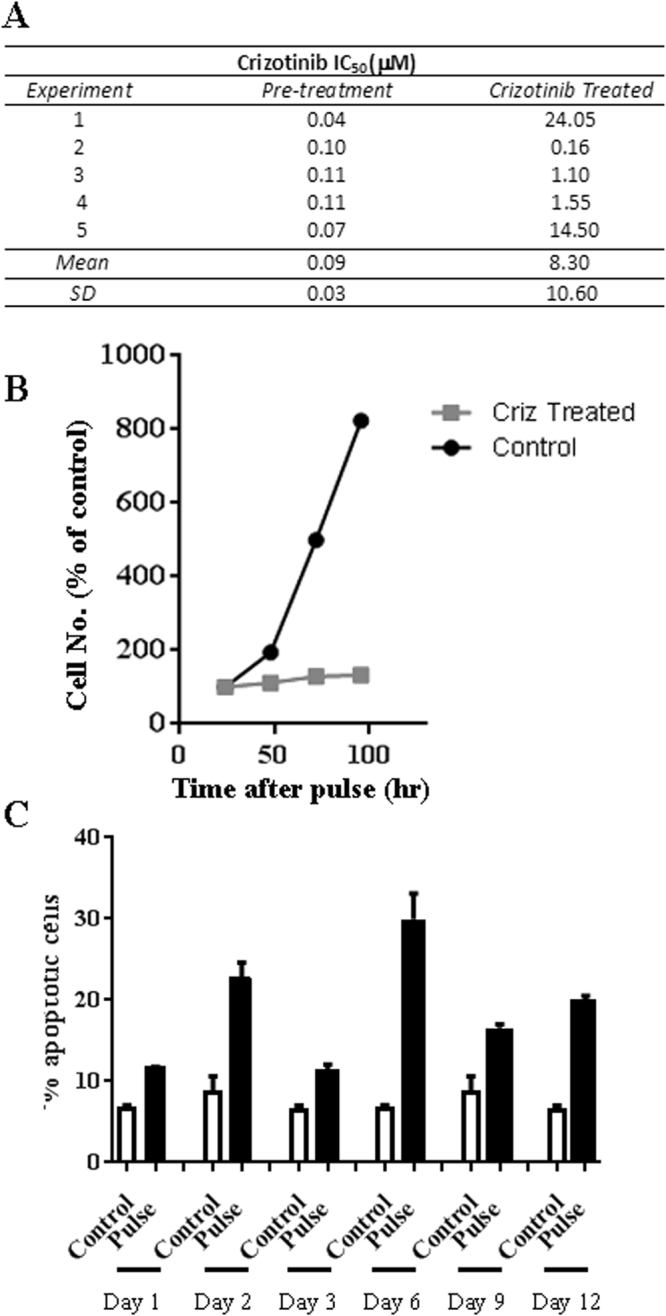
Effect of 24 hr exposure of H3122 cells to crizotinib (10 μM). (**A**) Change in IC_50_ for crizotinib cytotoxicity before and 12 days after treatment in five separate experiments. (**B**) Growth rate for H3122 cells 12 days after crizotinib treatment – data shown are for the first experiment in “A”. Pulse refers to the transient crizotinib exposure. (**C**) Apoptosis in crizotinib “pulsed” cells up to 12 days after treatment (black bars) compared to control/untreated cells (white bars).

We then characterised cells that had been transiently exposure to crizotinib in order to select for crizotinib resistant cells. In five independent experiments, following 24 hour exposure to crizotinib (10 μM) the IC_50_ for crizotinib cytotoxicity had increased when the cells were assessed 12 days later. Results were highly variable, consistent with clonal cell selection cells (Fig. 2A). By contrast, the ALK-negative NSCLC cell line A549 showed only a 4.3-fold increase in the IC_50_ (Supplementary material, Fig. S1). Cell proliferation was investigated in both crizotinib-treated and non-treated control cells; H3122 cells increased in number only slightly through this period (Fig. 2B) whereas crizotinib treated A549 cells grew at a similar rate to untreated cells (Fig. S1). Further analysis showed that transient crizotinib treatment had caused significant increase in the subG_1_ apoptotic fraction of cells up to 12 days after crizotinib treatment compared to control cells (p < 0.05, Fig. 2C).

**Figure 3:**
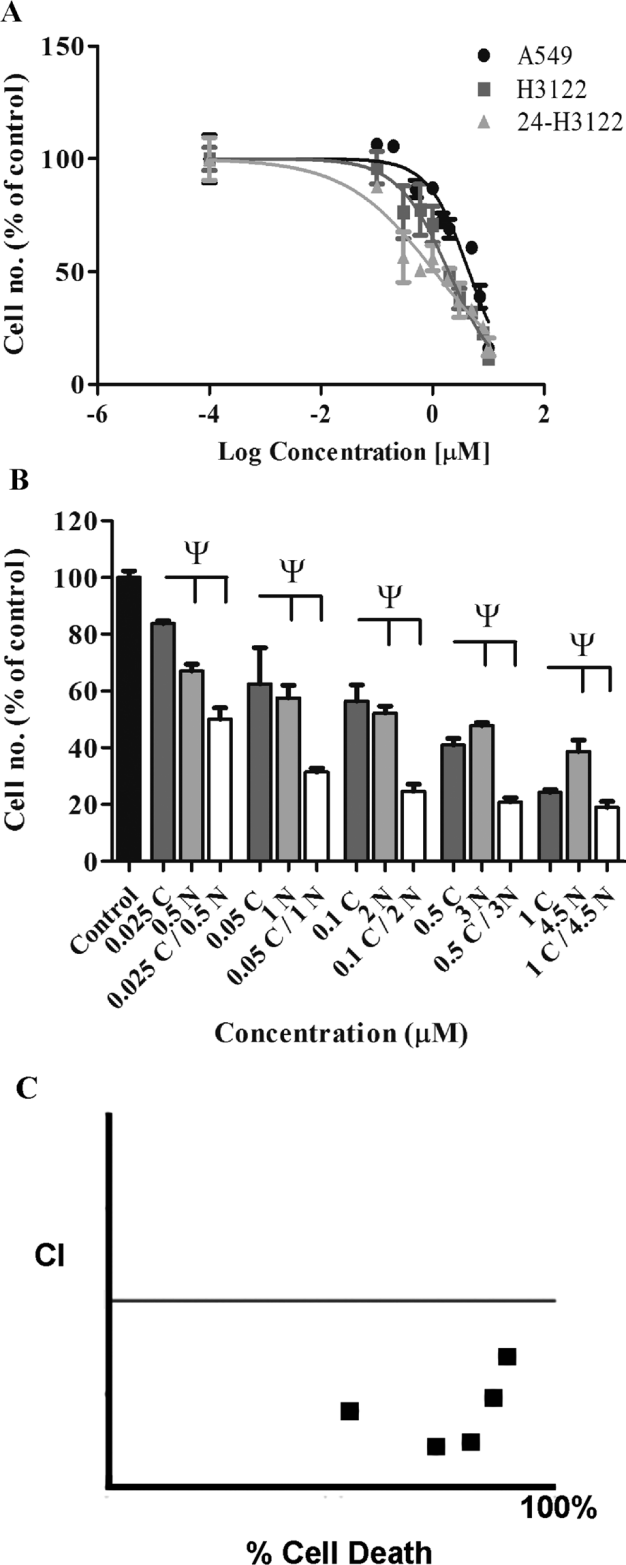
Effect of 24 hr exposure of NSCLC cells to crizotinib (10 μM) on drug cytotoxicity at 12 days post-treatment. (**A**) Cytoxicity for IGF-1R inhibitor NVP-AEW541 on ALK-negative A549 cells, ALK-positive H3122 cells, and crizotinib-exposed cells (24-H3122). (**B**) Cytotoxicity of combination treatment by crizotinib and NVP-AEW541 on 24-H3122 cells. (**C**) Combination index plot for drug combinations in “B”. Conventions are as for Fig. 1.

In contrast to crizotinib, the cytotoxicity for IGF-1R inhibition from NVP treatment was not significantly different between ALK-negative A549 cells and ALK-positive H3122 cells (p > 0.05, Fig. 3A). The H3122 cells treated with crizotinib were slightly *more* sensitive to NVP than untreated cells 12 days after crizotinib treatment (p < 0.001, Fig. 3A). Synergistic toxicity from combining crizotinib with NVP was retained in the crizotinib treated cells at 12 days after treatment (Fig. 3B,C).

**Figure 4:**
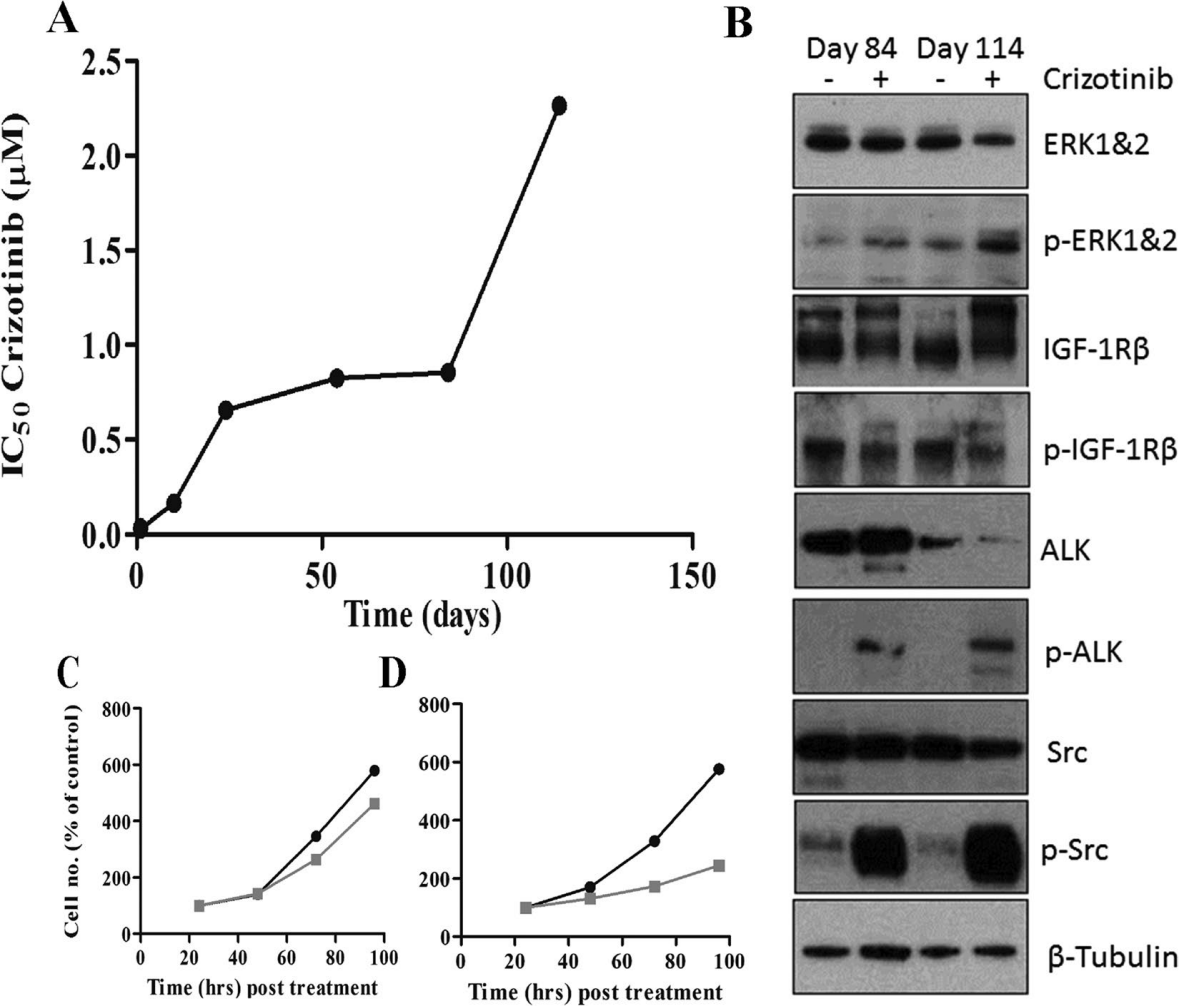
Characterisation of H3122 cells with acquired crizotinib resistance (CR-H3122). (**A**) Change in IC_50_ values for cytotoxicity during the course of acquisition of crizotinib resistance. (**B**) Tyrosine kinase expression and activation (phosphorylation) in CR-H3122 cells (+) compared with H3122 cells (−) at 84 and 114 days following initiation of maintenance of cells in crizotinib (see main text for details). Each antibody was performed on separate blots, as delineated with dividing lines. (**C**) Growth rate of CR-H3122 cells compared to H3122 cells at 84 days and 114 Days (**D**).

Crizotinib-resistant H3122 cells (CR-H3122) were generated by incubating the cells over a period 114 days. Cells were exposed to increasing concentrations of crizotinib over several weeks up to a maximum of 1 μM. Cells were examined at various times during this chronic exposure to crizotinib, with an increase in the IC_50_ detected as early as 10 days following treatment initiation (IC_50_ initially 0.03 μM increased to 0.16 μM) accompanied with a decrease in the proliferation rate compared to control cells (Fig. 4A). By day 114 the IC_50_ for crizotinib cytoxicity had increased to over 2.3 μM, a greater than 20-fold increase over parental H3122 cells. Western blot showed that compared to control H3122 cells (Fig. 4B), the crizotinib resistant CR-H3122 cells had an increase in ALK and ERK phosphorylation at days 84 and 112, with a very high increase in SRC phosphorylation. By contrast, IGF-1R phosphorylation was moderately reduced (see supplementary material for further details). The growth rate of the crizotinib resistant cells was initially greatly reduced (data not shown) but increased by days 84 and 114, although still slower than the parental H3122 cell line (Fig. 4C,D).

**Figure 5:**
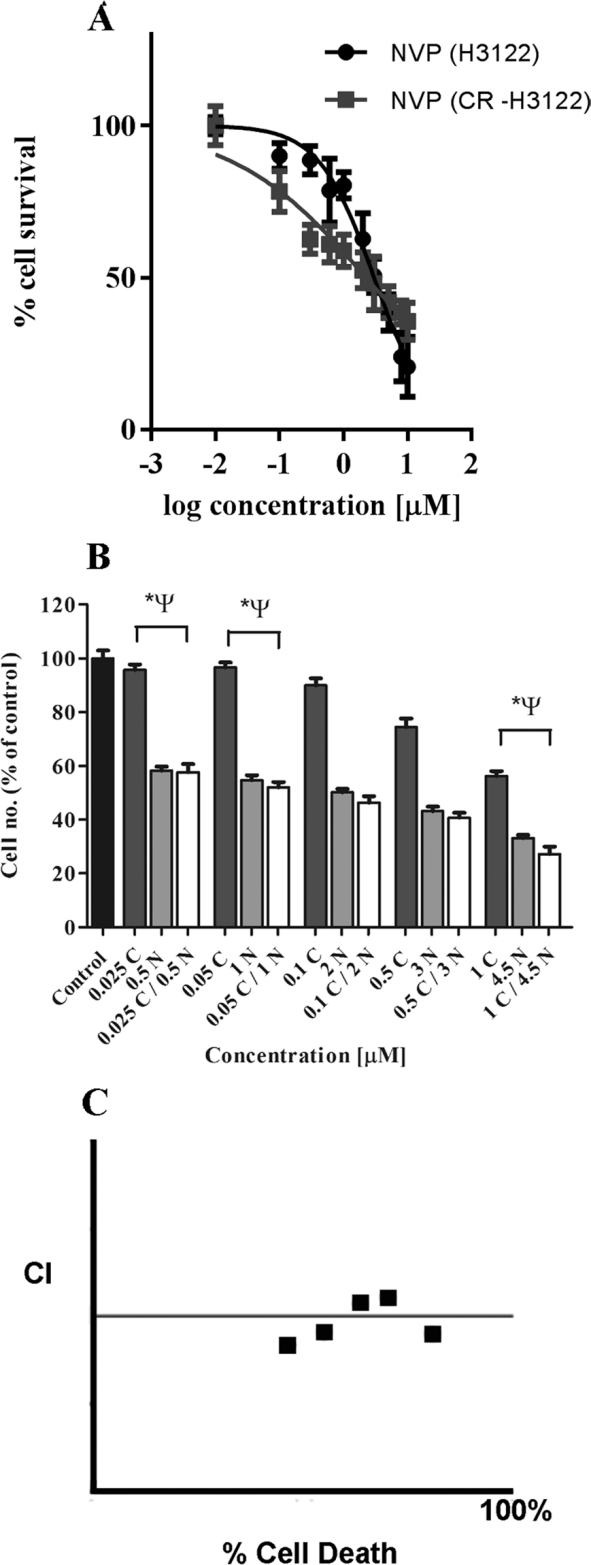
Effect of acquired resistance to crizotinib on drug cytotoxicity. (**A**) Cytoxicity for IGF-1R inhibitor NVP-AEW541 (NVP) H3122 and CR-H3122 cells. (**B**) Cytotoxicity of combination treatment by crizotinib and NVP-AEW541 on CW-H3122 cells. (**C**) Combination index plot for drug combinations in “B”. Conventions are as for Figs 1 and 3.

The difference between IC_50_ values that we measured for NVP in CR-H3122 cells was not significantly different from that in parental H3122 cells (p > 0.05, Fig. 5A). Therefore resistance to ALK inhibition was not correlated with resistance to IGF-1R inhibition in our model. When crizotinib was combined with NVP, CR-H3122 cells appeared to be re-sensitized to crizotinib (Fig. 5B). However, closer examination of the data revealed that combining NVP with crizotinib caused no more cell death than NVP alone, and that the combination of the two drugs did not act synergistically to kill cells (Fig. 5C).

**Figure 6:**
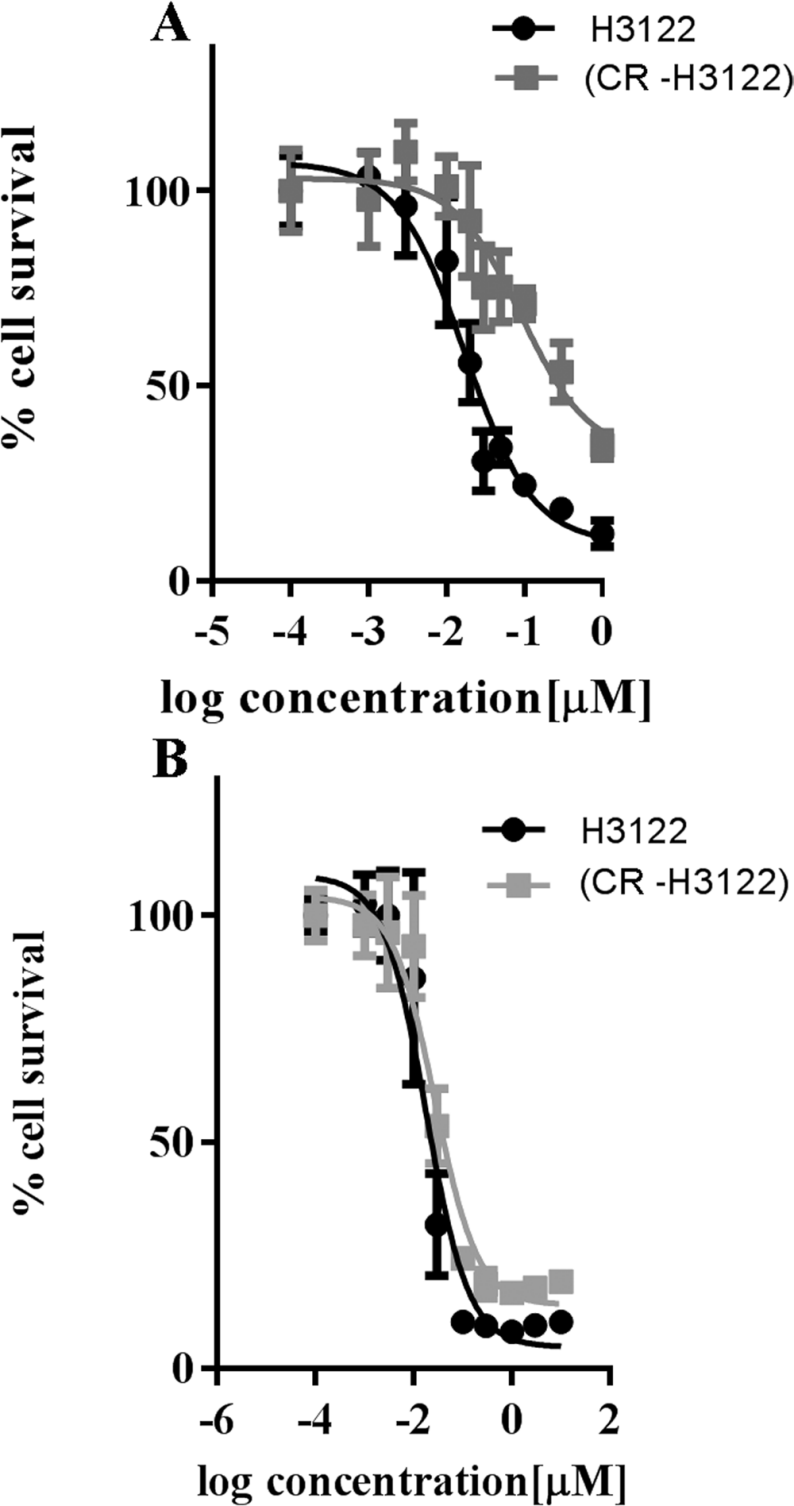
Effect of acquired resistance to crizotinib on cytotoxicity (**A**) Cytoxicity of ceritinib in H3122 and CW-H3122 cells. (**B**) Cytoxicity of AZD3463 in H3122 and CR-H3122 cells. Conventions are as for Fig. 1.

In contrast to Lovly *et al*.^14^ the CR-H3122 cells had also developed significant cross resistance to the dual ALK/IGF-1R inhibitor ceritinib; an IC_50_ shift from 0.01 in H3122 cells to 0.09 in crizotinib-resistant cells (p < 0.001, Fig. 6A). However, the CR-H3122 cells *did not* develop resistance to a different dual ALK/IGF-1R inhibitor, AZD3463; for which cytotoxicity IC_50_ values for AZD3463 evaluated in H3122 cells and in CR-H3122 cells were 0.03 μM and 0.05 μM respectively, an insignificant difference (Fig. 6B, p > 0.05).

## Discussion

We successfully replicated the demonstration by Lovly *et al*.^14^ that crizotinib combines synergistically with an IGF-1R inhibitor to kill ALK^+^ lung adenocarcinoma cells. We further demonstrated that this synergy was retained for up to 12 days after 24 hr exposure to 10 μM crizotinib. However, in contrast to Lovly *et al*.^14^ we found that in our model of *acquired* resistance to crizotinib, combining crizotinib with IGF-1R inhibition was no longer synergistic; specifically, adding crizotinib to the IGF-1R inhibitor did not increase cytotoxicity over that obtained by IGF-1R inhibition alone (Fig. 5). However, and most importantly, our crizotinib resistant cells did not develop cross resistance to IGF-1R inhibition. Therefore, ALK and IGF-1R are independent drug targets according to the criterion developed by Bozic and Nowak^19,20^ in their models of drug combination to overcome cancer resistance, and our results are consistent with their proposal that combination therapy will be more effective than monotherapy. However, although we found that (contrasting with Lovly *et al*.^14^) cross resistance had developed in CR-H3122 cells to the dual ALK/IGF-1R inhibitor ceritinib, we did observe a moderate sensitization to IGF-1R inhibition in our model of acquired crizotinib resistance, which may support the use of such a strategy as a monotherapy in crizotinib resistant patients.

An interesting result in our investigations was the lack of cross resistance to another dual ALK/IGF-1R inhibitor, AZD3463, in our crizotinib-resistant cells. We propose that since the cells did not seem to be susceptible to synergistic ALK/IGF-1R cytotoxicity (above) then this may be due to altered binding characteristics to ALK by this drug compared with both crizotinib and ceritinib. Further analysis of these cells will be required to establish the presence of ALK mutations, and whether these mutations confer differential sensitivity to ceritinib and AZD3463.

Alterations in kinase phosphorylation in our resistant cells compared with control cells are consistent with our drug cytotoxicity results. In contrast to the clinical observations made Lovly *et al*.^14^ we did not see an increase in IGF-1R phosphorylation in the crizotinib resistant cells. However, we also investigated the MAPK pathway because this has been strongly argued by Hrustanovic *et al*.^21^ to be the dominant growth and survival pathway in ALK-mutated lung cancer. Our findings were consistent with this hypothesis. Notably, the most dramatic change we measured was in SRC phosphorylation, which is also consistent with the clinical observations of Crystal *et al*.^10^ that SRC activating mutations conferred ALK inhibitor resistance in patients. Ongoing work will determine if SRC inhibitors will resensitize these cells to crizotinib toxicity.

The practical feasibility of combination ALK/IGF-1R inhibition as a clinical strategy has been given a boost by recent research into the IGF-1R inhibitor AXL1717 in advanced non-small cell lung cancer. Early results have indicated that as a monotherapy AX1717 is as effective as existing chemotherapy and with less toxicity^22–24^. Moreover, the role of IGF-1R activation in non-small lung cancer prognosis has been very recently confirmed in a study of 326 NSCLC patients^25^.

In conclusion, our results corroborate the hypothesis that combination ALK and IGF-1R inhibition overcomes primary crizotinib resistance in ALK^+^ lung cancer cells, but does not necessarily overcome *acquired* crizotinib resistance. Our results also support the hypothesis that ALK and IGF-1R are independent druggable targets in ALK-positive lung cancer.

## Electronic supplementary material

Supplementary Material
