# Supplementary material for: ALK and IGF-1R as independent targets in crizotinib resistant lung cancer
Source: Sci Rep. 2017 Oct 24;7:13955. doi: 10.1038/s41598-017-14289-w (PMC5654778; doi:10.1038/s41598-017-14289-w)
Supplement: Supplementary file 1 — Supplementary Material [file 41598_2017_14289_MOESM1_ESM.pdf]

Supplementary Material

ALK and IGF-1R as independent targets in crizotinib resistant lung cancer

Christabel Wilson[1], Mhairi Nimick[1], Hayley Nehoff[1], John C. Ashton[1*]

1. Department of Pharmacology & Toxicology, Otago School of Biomedical Sciences, University of Otago, Dunedin, New Zealand.

*corresponding author +6434793040 john.ashton@otago.ac.nz

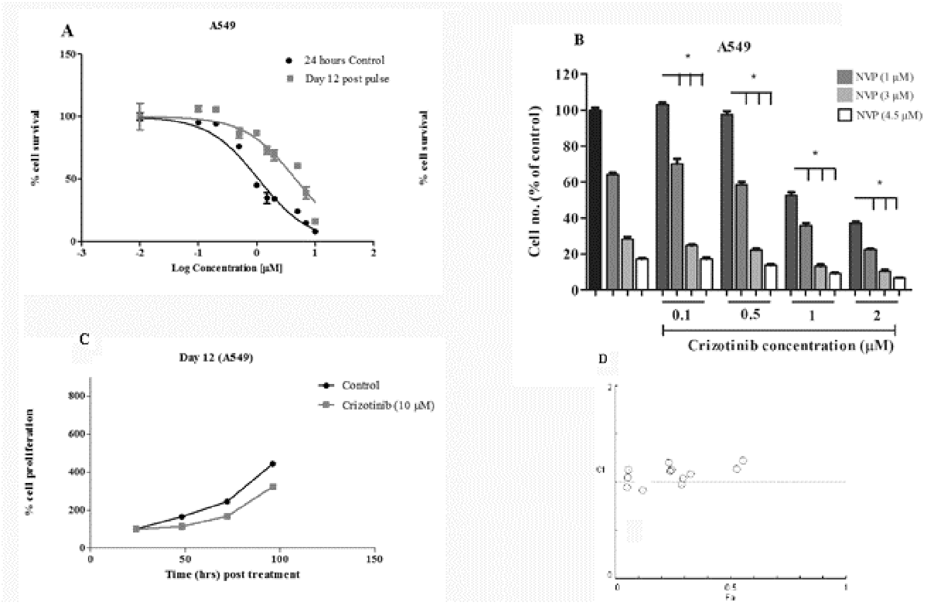

Figure S1. In contrast to ALK-positive H3122 lung cancer cells, ALK-negative A549 lung cancer cells had only a small change in IC50 for crizotinib cytotoxicity 12 days after exposure to crizotinib (see main text for details) (A). Combination ALK/IGF-1R inhibition had an additive rather than synergistic effect on these cells (B,D), and cell growth was little affected by prior crizotinib exposure (C). Conventions are as for main figures.

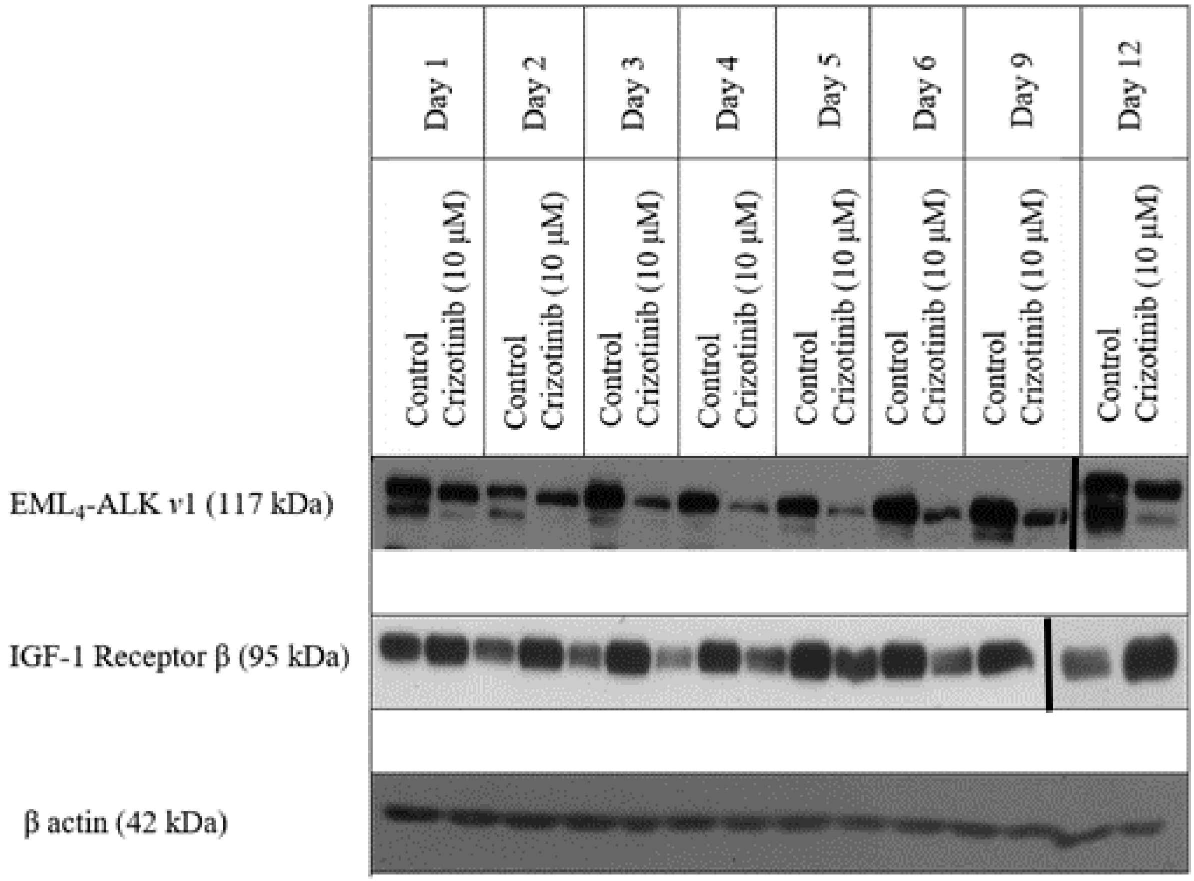

Figure S2. Changes in total ALK and IGF-1R expression cells treated for 24 hours with 10 μM crizotinib (CR) over 12 subsequent days compared to untreated cells (0). There is a notable transient decrease in ALK and increase in IGF-1R. Vertical black lines indicate where separate blots have been combined.

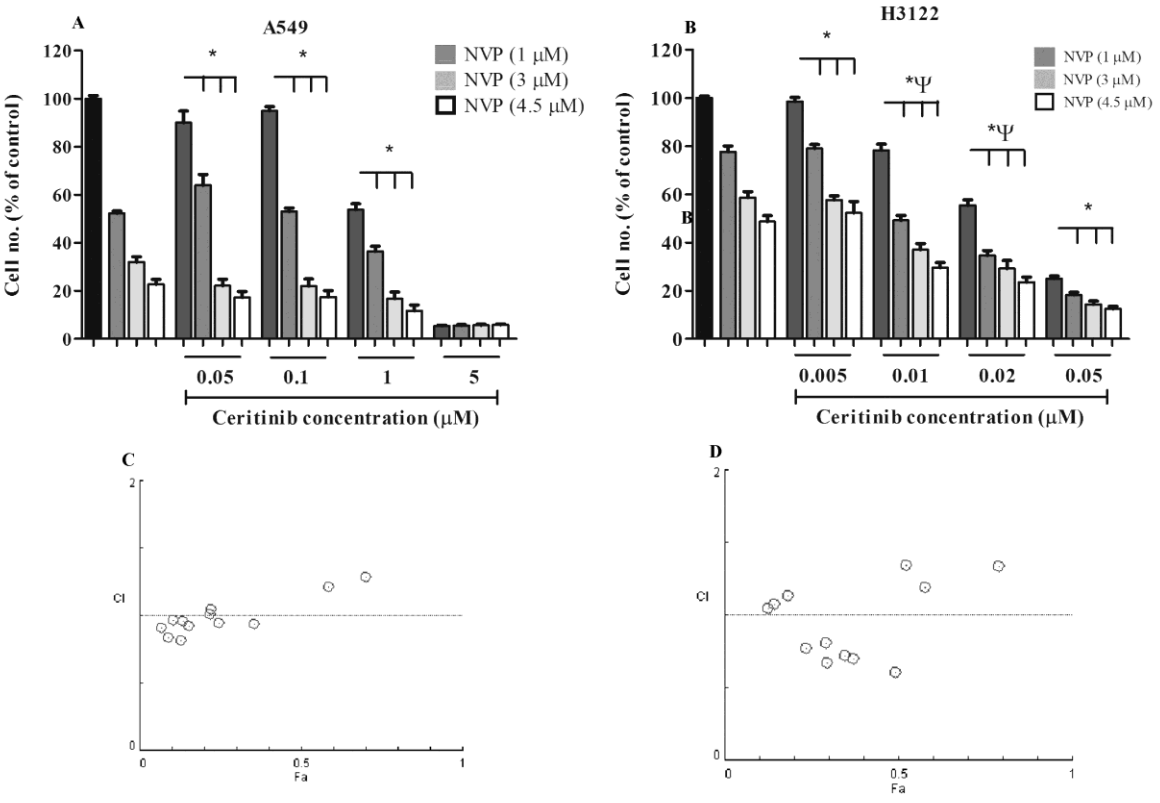

Figure S3. Cytoxicity of combination treatment with ceritinib and NVP-AEW54 on A549 (A, C) and H1322 cells (B,D). Conventions are as for main text.

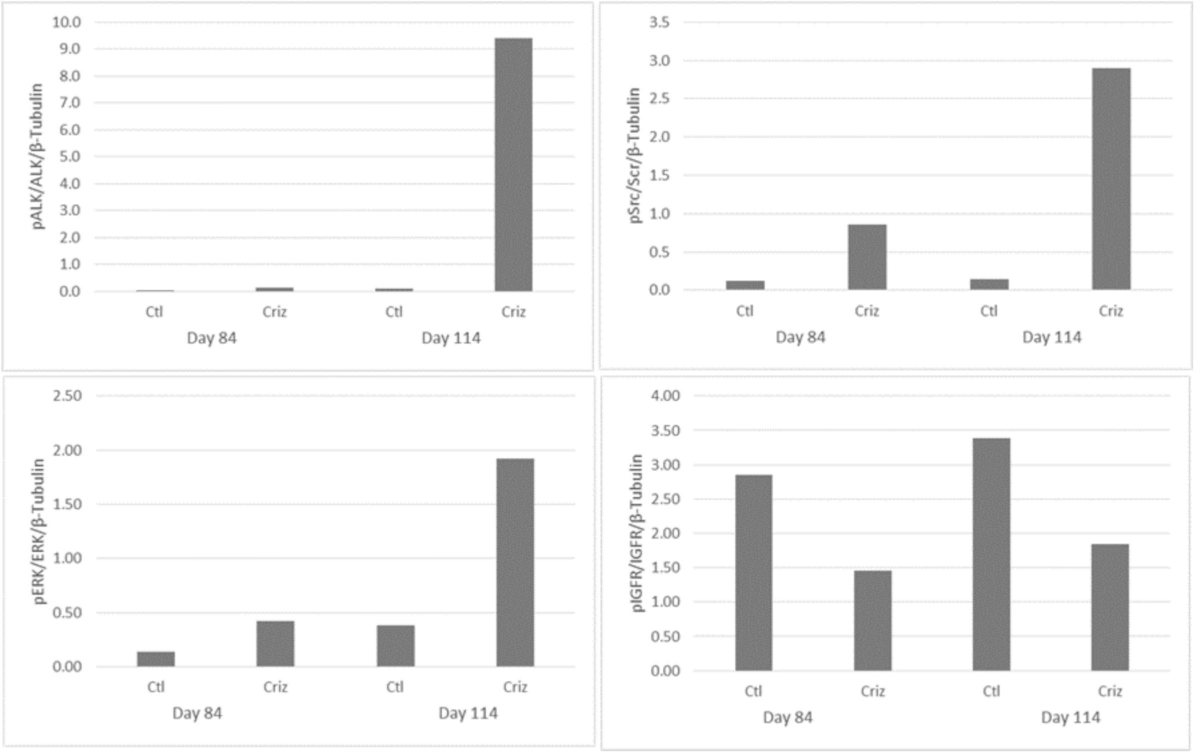

Figure S4. Quantification of kinase activation using densitometry for the Western blots shown in Fig. 4.

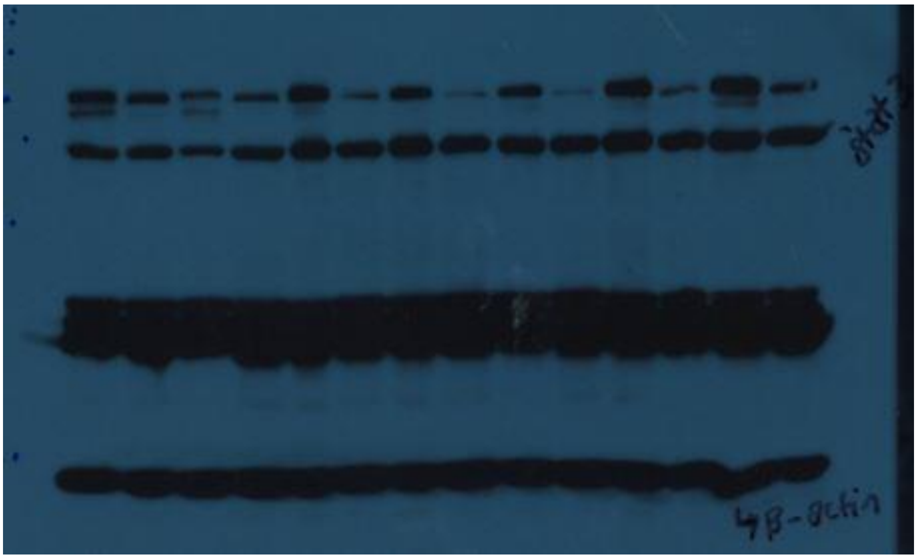

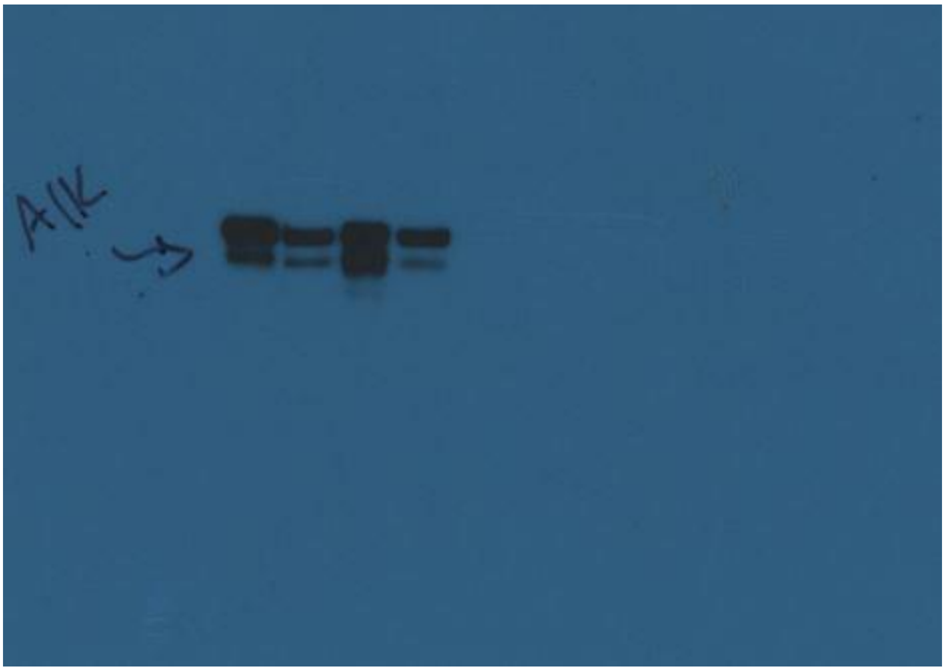

Figure S5. Raw images of Western blots for ALK labelling used in figure S2. First was probed with four separate primary antibodies (from top to bottom: ALK, Stat3, ERK, and beta-actin). The ALK band was used in figure S2. The second blot shows the day 12 samples from figure S2 (in duplicate on this blot, one taken for figure S2) where only the ALK antibody was used.

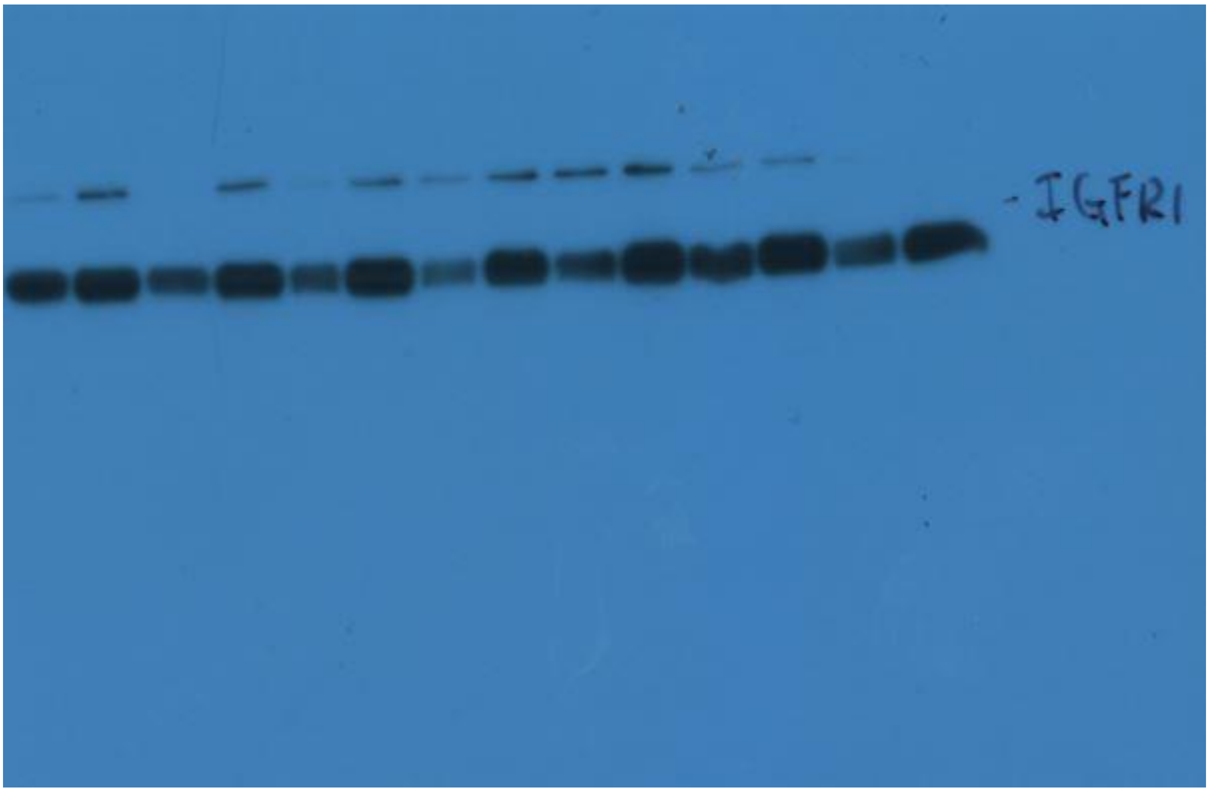

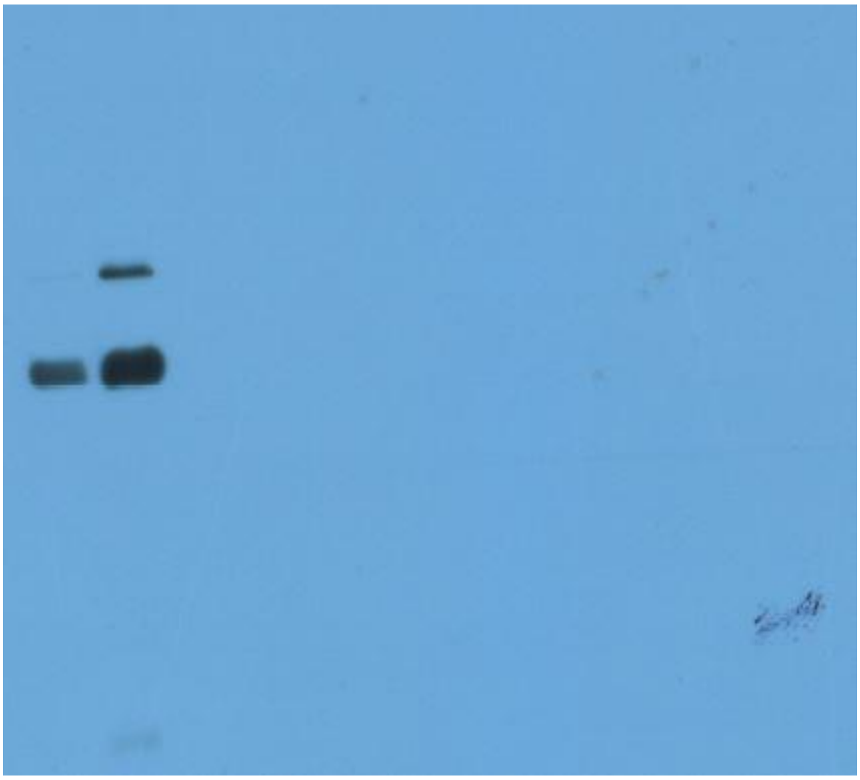

Figure S6. Raw images of Western blots for IGF1-R labelling used in figure S2. The first blot shows days 1 to 9, and the second shows day 12.

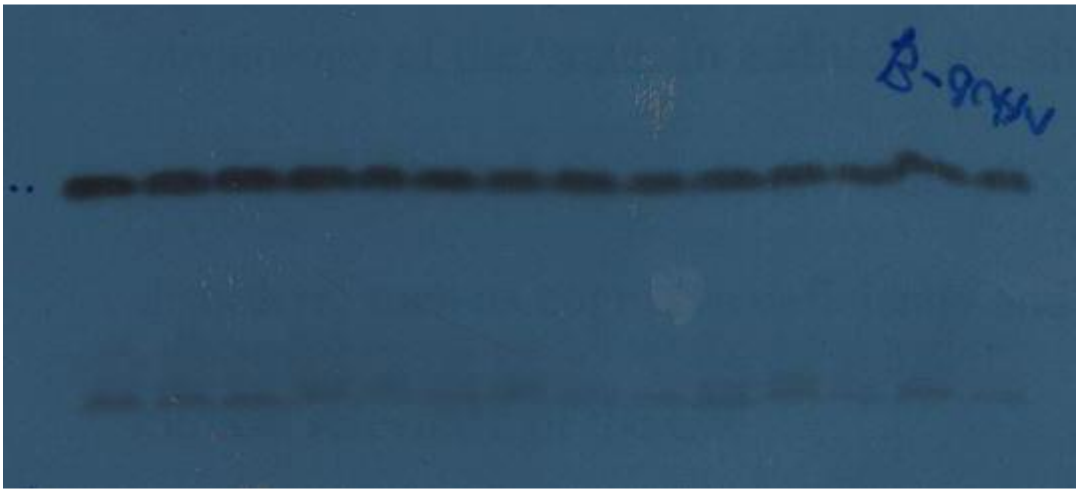

Figure S7. Raw image of beta-actin labelling shown in figure S2, at a lower exposure than shown in in figure S5.
